# A Synergistic, Balanced Antioxidant Cocktail, Protects Aging Rats from Insulin Resistance and Absence of Meal-Induced Insulin Sensitization (AMIS) Syndrome

**DOI:** 10.3390/molecules20010669

**Published:** 2015-01-06

**Authors:** Hui Helen Wang, Kawshik K. Chowdhury, W. Wayne Lautt

**Affiliations:** Department of Pharmacology & Therapeutics, College of Medicine, Faculty of Health Sciences, University of Manitoba, A224-753 McDermot Avenue, Winnipeg, MB R3E 0T6, Canada; E-Mails: kawshik88@yahoo.com (K.K.C.); wlautt@cc.umanitoba.ca (W.W.L.)

**Keywords:** HISS-dependent insulin resistance (HDIR), Meal-induced Insulin Sensitization (MIS), Absence of Meal-induced Insulin Sensitization (AMIS), Hepatic Insulin Sensitizing Substance (HISS), *S*-adenosylmethionine, vitamins E and C (SAMEC)

## Abstract

A series of *in vivo* and *in vitro* studies using animal and human models in the past 15 years have demonstrated that approximately 55% (~66% in humans) of the glucose disposal effect of an i.v. injection of insulin in the fed state is dependent on the action of a second hormone, hepatic insulin sensitizing substance (HISS), which is released from the liver and stimulates glucose uptake in muscle, heart and kidneys. Sensitization of the insulin response by a meal through release of HISS is called meal-induced insulin sensitization (MIS). Absence of HISS action results in postprandial hyperglycemia, hyperinsulinemia, hyperlipidemia, adiposity, increased free radical stress and a cluster of progressive metabolic and cardiovascular dysfunctions referred to as the AMIS (absence of meal-induced insulin sensitization) syndrome. Reduced HISS release accounts for the insulin resistance that occurs with aging and is made worse by physical inactivity and diets high in sucrose or fat. This brief review provides an update of major metabolic disturbances associated with aging due to reduction of HISS release, and the protection against these pathological changes in aging animals using a balanced synergistic antioxidant cocktail SAMEC (*S*-adenosylmethionine, vitamins E and C). The synergy amongst the components is consistent with the known benefits of antioxidants supplied by a mixed diet and acting through diverse mechanisms. Using only three constituents, SAMEC appears suitable as an antioxidant specifically targeting the AMIS syndrome.

## 1. Introduction

### Aging and Age-Related Metabolic Dysfunctions: The Role of HISS and AMIS

Aging is often, and unnecessarily, associated with predictable pathologies and reduced homeostatic capacity in multiple organs. The systems that shut down often do so gradually, and are lifestyle related. Aging is associated with a wide range of diseases and metabolic disorders, such as reduced vascular and cardiac health, obesity, and diabetes, and aging itself has characteristics of a disease with accumulated cellular starvation, dehydration and damage [1,2,3,4]. Oxygenated radicals have a role in gene expression, cell oxidative injuries and cytotoxic activity of the immune system [5]. Antioxidants, especially those in mixed diets, are associated with lowering oxidative stress, DNA damage, malignant transformation, and other parameters of cell damage *in vitro*, and lowering the incidence of certain types of cancer and degenerative diseases [6]. We propose that many age-related metabolic dysfunctions are the result of a postprandial metabolic deficiency, secondary to absence of the actions of a Hepatic Insulin Sensitizing Substance, HISS.

The relationship of HISS to the metabolic dysfunctions that occur with normal aging became clear, as a series of studies determined that the age of the control groups (*vs*. treatment groups) was strongly associated with HISS action [7,8,9]. In this review we summarize the studies related to the pathophysiology of HISS and the consequences of reduced HISS action. We show that the detrimental effects of sucrose supplement and beneficial effects of exercise and antioxidants all act through affecting HISS [10,11,12].

An antioxidant cocktail combining *S*-adenosylmethionine, vitamins E and C proved to be synergistically effective, whereas the individual components were ineffective [13]. The synergistic interaction of antioxidants was unexpected and may account for the effectiveness of antioxidants consumed in foods as a mixture of a wide range of antioxidant molecules, whereas clinical trials of single or double ingredients have generally been unimpressive. A balanced antioxidant dietary supplement was originally developed as a research tool to study the physiology and pathophysiology of HISS [13]. Understanding the physiology of HISS allows early diagnosis and intervention for many of the metabolic and cardiovascular dysfunctions associated with age. These studies show a very significant role for balanced antioxidant management to prevent some of the most debilitating health issues in the elderly.

## 2. Summary of the HISS Story

In the normal healthy state, feeding results in a rapid meal-induced insulin sensitization (MIS) of glucose uptake into various cell types in peripheral tissues [14,15,16]. MIS results from insulin, acting in the presence of feeding signals delivered to the liver, causing the release of a hepatic insulin sensitizing substance (HISS). HISS acts selectively on skeletal muscle, heart and kidneys, but not liver or adipose tissues, to stimulate glucose uptake [17,18,19]. HISS action accounts for approximately 55% of the total glucose disposal response to a pulse of insulin in the fed state of rats [15,20], and ~66% in humans [21]. In order for insulin to cause the release of HISS, two permissive feeding signals are needed [17]. One signal is delivered via hepatic parasympathetic nerves acting on muscarinic receptors, and subsequent activation of nitric oxide synthase and elevated cGMP [22,23]. The other is a chemical signal seen as an approximately 40% elevation in hepatic glutathione (GSH) [24,25,26]. Both signals are needed, as either signal alone is not sufficient to activate the HISS pathway [26,27]. The feeding signals decrease with the duration of fasting until by 24 h HISS release is minor or absent. The MIS process has now been demonstrated in mice, rats, guinea pigs, cats, dogs, and humans by independent laboratories [28].

### HISS-Dependent Insulin Resistance (HDIR)

The development and consequences of HDIR are most dramatic following meal consumption, when glucose is being absorbed from the consumed food to enter the bloodstream to be rapidly and efficiently stored [29]. Glucose in the blood causes pulsatile release of insulin, which reaches the liver where reflexely activated parasympathetic nerves and rapidly elevated hepatic GSH serve as permissive regulators to allow pulses of insulin to stimulate pulsatile releases of HISS [29]. HISS enters the bloodstream and stimulates glucose uptake primarily into skeletal muscle [30,31], doubling the glucose disposal effect of a pulse of insulin. Absence of HISS action (HDIR) results in a major shift in storage of nutrient energy from glycogen in muscle to fat.

HDIR is physiologically and appropriately produced in the fasted state when the hypoglycemic effect of HISS would not be advantageous. HISS-dependent insulin resistance can also be induced in the fed state when HISS action is blocked by various approaches interfering with the pathways of either of the two permissive regulatory signals required for the release of HISS. The neural signal can be blocked by surgical parasympathetic denervation of the liver [31,32], blockade of hepatic cholinergic muscarinic receptors [32,33], blockade of hepatic nitric oxide production [22,34,35], or blockade of hepatic cyclooxygenase [36]. The GSH signal can be blocked by inhibiting production [25,27], or acutely depleting the GSH using acute alcohol [28,37] and possibly other drugs that rapidly deplete GSH.

Various animal models of HDIR have been established for study of the relationship and control mechanisms of insulin resistance through regulation of the HISS activation pathway. These models include high-sucrose or fat diets, hepatotoxins, chronic liver diseases, spontaneously hypertensive or obese animal strains, aging, acute hemorrhage [13,26,38,39,40,41,42,43,44], fetal alcohol exposure [36,45,46] and late gestation (unpublished personal communication).

## 3. The AMIS Syndrome

Absence of meal-induced insulin sensitization (AMIS) results from diminished HISS action following a meal. A healthy MIS involves the actions of insulin and HISS, with the result being tight glycemic control and storage of nutrient energy primarily as glycogen in muscle. Acute AMIS results in postprandial hyperglycemia with compensatory hyperinsulinemia. The elevated insulin acts primarily on the liver and adipose tissue and results in hyperlipidemia and elevated free radical production and a shift in nutrient storage from glycogen to fat. HISS also accounts for the vasodilation in skeletal muscle associated with insulin, and AMIS is associated with a lack of a dilator response to insulin administration [47]. Chronic AMIS results in a progressive, predictable series of metabolic and cardiovascular dysfunctions that can be increased or decreased by manipulating HISS release.

We suggest that the initiating metabolic defect that leads progressively to the metabolic syndrome, diabetes, and multiple organ failure is a postprandial defect in glucose sequestration that results in nutrient energy being shifted from normal storage as glycogen in skeletal muscle to production and storage of lipids, and postprandial hyperglycemia, hyperinsulinemia, hyperlipidemia and increased reactive oxidative stress ([7,9], see table 1 in ref [7]). The collective signs and symptoms associated with AMIS is referred to as the AMIS syndrome [7]. Several of the studies unraveling the chronology of the AMIS syndrome used aging as one parameter to manipulate HISS action.

Resistance to the direct action of insulin on glucose uptake is demonstrable in the fasted state or after HISS release has been blocked by any means. In the various models of HDIR, the direct action of insulin is not impaired until a late stage of the AMIS syndrome, well after adiposity and cardiovascular dysfunctions have occurred.

## 4. Aging, HISS and HDIR

### 4.1. Aging and HISS Release

Insulin resistance refers to a condition where cells in the body become resistant to the effects of insulin, which means the normal response to a given amount of insulin is reduced. Aging is frequently associated with accumulation of body fat (the first symptom of the AMIS syndrome [17]) and altered body composition. Mid-aged and elderly populations tend to be more susceptible than younger populations to developing insulin resistance and type 2 diabetes [48]. Mechanisms underlying age-related development of obesity and type 2 diabetes are still generally considered unknown in the literature [49]. Insulin resistance has been considered to be the key etiologic defect that defines the metabolic syndrome [50], and obesity together with other multifactorial conditions (such as inflammation, lipid metabolism abnormality) has been proposed as the cause of insulin resistance [51]. Obesity as an early component of the AMIS syndrome has recently been reviewed [17].

In attempting to explain why aging is related to insulin resistance, obesity and type 2 diabetes [49,52,53], various studies on interdependency and reciprocal interactions between chronic inflammation, obesity-induced insulin resistance and their effects on accelerating the aging process, as well as age-related impairment of pancreatic beta-cell function have been reported [53,54,55]. However, the impact of aging on HISS release provides compelling evidence and a direct answer to the question.

### 4.2. The Aging Model of HDIR

**Figure 1:**
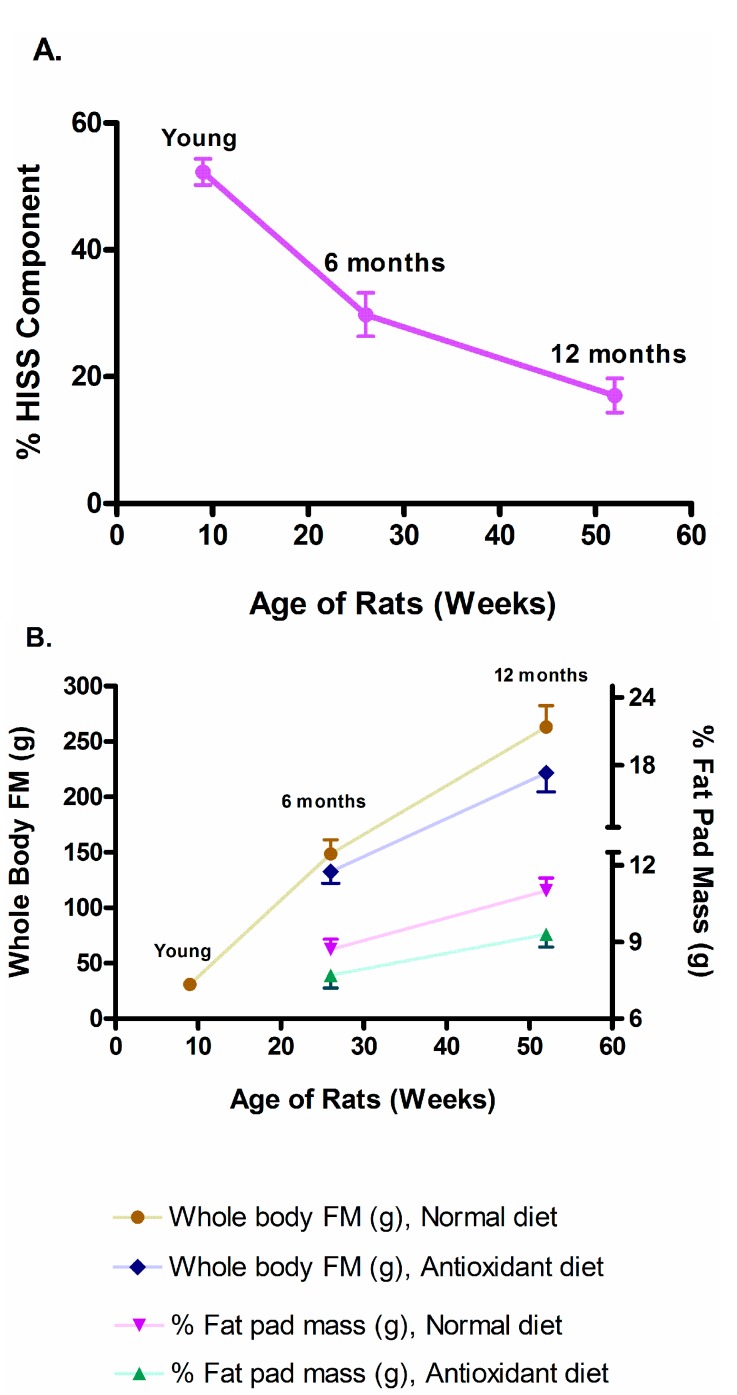
(**A**) The percentage (%) of HISS component decreased significantly as rats age increased from 9 to 52 weeks. *p* < 0.01: Young *vs*. 6 month, and 6 *vs*. 12 month groups [41]. (**B**) HISS action correlated negatively with whole body adiposity and all regional fat depots as rats age increased. Whole body fat mass (FM) and % Fat pad mass: *p* < 0.05: All 26 and 52 week old *vs*. young rats in the same group; 26 *vs*. 52 week old rats with normal diet. % Fat pad mass: *p* < 0.05: 52 week old rats with normal *vs*. antioxidant diet ([41] Table 1).

Using an *in vivo* euglycemic clamp, the rapid insulin sensitivity test (RIST) [56], Lautt *et al.* tested male Sprague Dawley rats at 9, 26, and 52 weeks of age to determine their dynamic response to insulin, which contains the HISS-dependent (HISS action) and HISS-independent components of insulin action (the sum of both components makes up the total insulin action on glucose disposal). In young rats, the HISS component accounted for 52.3% ± 2.1% of the response to a bolus of insulin, which decreased to 29.8% ± 3.4% at 6 months (26 weeks) and 17.0% ± 2.7% at 12 months (52 weeks) of age (Figure 1A). In addition, HISS action correlated negatively with whole body adiposity and all regional fat depots ([41], Figure 1B).

Ribeiro *et al.* demonstrated that total insulin action decreased gradually from 6 to 52 weeks of age in fed or fasted, male and female Wistar rats. The HISS-independent component of insulin action decreased until 9 weeks of age and remained unchanged thereafter, whereas the HISS-dependent component decreased from 9 weeks of age throughout aging [42]. These studies demonstrate that the progressive decrease in HISS action accounts for the gradual decrease of the response to insulin associated with aging [42]. Decreased parasympathetic nervous activity occurs with age [3,57,58,59,60] and could be the fundamental pathology of age-related decrease of HISS release, resulting in many of the pathologies associated with aging. The HDIR induced by eliminating the parasympathetic signal (e.g., surgical denervation of the liver, blockade of hepatic muscarinic receptors, hepatic nitric oxide synthase, or hepatic cyclooxygenase) provides the direct support of parasympathetic nerve function being essential for hepatic HISS release in response to a bolus of insulin. The fact that a rat made HDIR using a sucrose supplement can have MIS fully restored using bethanechol (that mimics parasympathetic feeding signal) plus *N*-acetylcysteine (that mimics GSH feeding signal) demonstrates that the peripheral cells remain responsive to HISS and the liver cells remain responsive to insulin. The ability to restore MIS using drugs that mimic the 2 feeding signals is consistent with the signals being permissive [26]. A number of protocols were added to the aging studies to potentiate and inhibit AMIS.

### 4.3. Cardiac Dysfunction

In addition to the metabolic disturbances, progressive development of AMIS in aging rats further resulted in deterioration in cardiac performance [8]. As predicted, postprandial insulin resistance, including hyperglycemia, hyperinsulinemia, hyperlipidemia and oxidative stress, are risk factors related to cardiovascular diseases (such as congestive heart failure, myocardial infarction, ventricular hypertrophy, endothelial nitric oxide impairment in systemic blood vessels and the heart, atherosclerosis, and hypercoagulability of blood) [61]. These risk factors for adverse cardiovascular events can be detected in the pre-diabetic subjects based upon the metabolic response to a test meal, even in the absence of altered fasting parameters or cellular resistance to the direct effect of insulin [61].

Aging rats (from 9–52 weeks) showed strong age-related cardiovascular impairment using *in vivo* left ventricular pressure-volume loop analysis. Progressive reduction was seen in the rate of systolic contraction and diastolic relaxation, ejection fraction, cardiac output and ventricular elasticity. Progressive elevations occurred in ventricular end diastolic pressure, arterial blood pressure and peripheral vascular resistance. These dysfunctions correlated with the degree of AMIS and were made worse by a sucrose-supplemented diet and were protected with a balanced, synergistic antioxidant cocktail [8]. HISS results in the vasodilation associated with insulin [47] and may play a role in the changes noted in peripheral vascular tone associated with chronic AMIS.

Healthy non-obese people were divided into three tertiles on the basis of their baseline steady-state plasma glucose concentrations. Using insulin resistance as a predictor of age-related hypertension, coronary heart disease, stroke, cancer, and type 2 diabetes, the study [48] demonstrated that approximately 1 out of 3 healthy individuals in the upper tertile of insulin resistance had developed an age-related clinical event, followed for an average of 6 years, whereas no clinical events were observed in the most insulin-sensitive tertile [48]. Subjects in the highest steady-state plasma glucose concentration tertile were older, all had significantly higher body mass index, plasma triglycerides, total cholesterol and low-density lipoprotein concentrations, as well as higher areas under the curves of plasma glucose and insulin concentrations in comparison with the low steady-state plasma glucose concentration tertile of the most insulin-sensitive individuals [48]. This study demonstrated an association of early metabolic defects, consistent with the AMIS syndrome, leading to cardiovascular abnormalities that become serious pathologies of clinical events at a later stage of life.

## 5. Antioxidant Protection of Aging Animals

### 5.1. Development of a Synergistic Balanced Antioxidant Cocktail SAMEC (S-adenosylmethionine, Vitamins E and C)

Aging is proposed to be associated with free radical accumulation and damage to mitochondria [62,63,64], as well as reduced capacity to scavenge reactive oxygen species [40,65,66]. While insulin resistance results in excess free radicals, reactive oxygen species are also proposed as a trigger for insulin resistance [67]. Ming *et al.* have recently shown that thioacetamide, a hepatotoxin known to produce liver damage by generation of reactive oxygen species, resulted in HDIR that can be prevented by a synergistic combination of antioxidants, which targets simultaneously the aqueous phase (vitamin C), the lipid phase (vitamin E), and the mitochondria (*S*-adenosylmethionine) [13]. Synergy was shown by the lack of efficacy of the individual components. This balanced combination of antioxidants also proved to have efficacy in protection of aging animals from all aspects of pathological changes tested in association with the development of the AMIS syndrome with aging. This balanced antioxidant cocktail is referred to as SAMEC for convenience [13].

### 5.2. SAMEC Protection from Acute Liver Injury

Thioacetamide results in liver damage through oxidative stress [68,69]. Ming *et al.* investigated the hepatic protective effects of *S*-adenosylmethionine (SAMe) combined with/without vitamins C and E in rats treated with intraperitoneal thioacidamide [13]. The hepatotoxin caused liver tissue injury, increased liver enzymes, and decreased HISS action. Treatment with SAMe alone or vitamins C + E (given 1 h later) slightly improved liver histology but not the changes in liver enzymes and insulin sensitivity. Combined treatment with SAMe plus vitamins C + E greatly protected the liver from tissue injury, decreased liver enzymes and increased insulin sensitivity in these rats [13].

### 5.3. SAMEC Protection in a Low-Dose Sucrose Supplemented Model of Accelerated Development of AMIS

Over one year of aging, rats showed a slow development of AMIS that was accelerated by addition of a limited volume of low-dose sucrose supplement (5% sucrose solution) to the diet. Provision of SAMEC in the chow attenuated the rate and extent of development of AMIS in both normal aging animals and in aging animals on the sucrose diet. Adiposity, as assessed from weighed regional fat masses and from bioelectrical impedance to estimate whole-body adiposity, correlated strongly with AMIS (r^2^ = 0.7–0.8). SAMEC completely compensated for the negative impact of the age plus sucrose supplement on meal-induced insulin sensitization and attenuated development of the associated dysfunctions [7,9,40].

### 5.4. Cardiac Protection Conferred by SAMEC

Aging rats showed impaired cardiac performance evaluated using a Millar pressure volume conductance catheter system [8]. Poor cardiac performance correlated closely with the development of AMIS. The sucrose diet enhanced aging-associated cardiac dysfunction. In contrast, antioxidant treatment prevented sucrose-induced cardiac dysfunction in the old group. SAMEC provided a protective effect on HISS action, which not only prevented the sucrose-induced HDIR but allowed it to remain close to the levels seen in the young control rat group. SAMEC led to significantly higher levels of HISS action than were seen in the age-matched controls or sucrose groups. In addition, several other parameters (fed and fasting glucose and insulin levels, mean arterial pressure, whole body adiposity, postprandial triglycerides and LDL/HDL cholesterol) were also protected. HDIR, accounting for AMIS, appears to result in a mechanistically based collection of dysfunctions, suggesting that treatment or prevention of HDIR is likely to have beneficial effect on the full AMIS “syndrome” including cardiac dysfunction and type 2 diabetes [8].

### 5.5. Physical Exercise and SAMEC Effects on AMIS

Chowdhury *et al.* examined the impact and interaction of physical exercise, diet and antioxidants on HISS action in meal-induced insulin sensitization (MIS [12,70]). These studies demonstrated that high fat or sugar supplemented diet reduces MIS, exercise elevates MIS, and antioxidants protect MIS against reductions associated with diet and age. Voluntary running-wheel exercise in aging rats improved insulin sensitivity through improved HISS-dependent glucose uptake in all age groups, whereas the direct action of insulin (HISS-independent component of insulin action) was minimally altered by age or exercise. The older animals and those on the sucrose supplement showed the greatest improvement in HISS action per unit of exercise. Body composition and metabolic parameters were beneficially changed in correlation with exercise-induced improvements in the HISS response [11]. In addition, the antioxidant cocktail, SAMEC, did not affect the benefits of exercise on insulin sensitivity [70].

## 6. Conclusions

**Figure 2:**
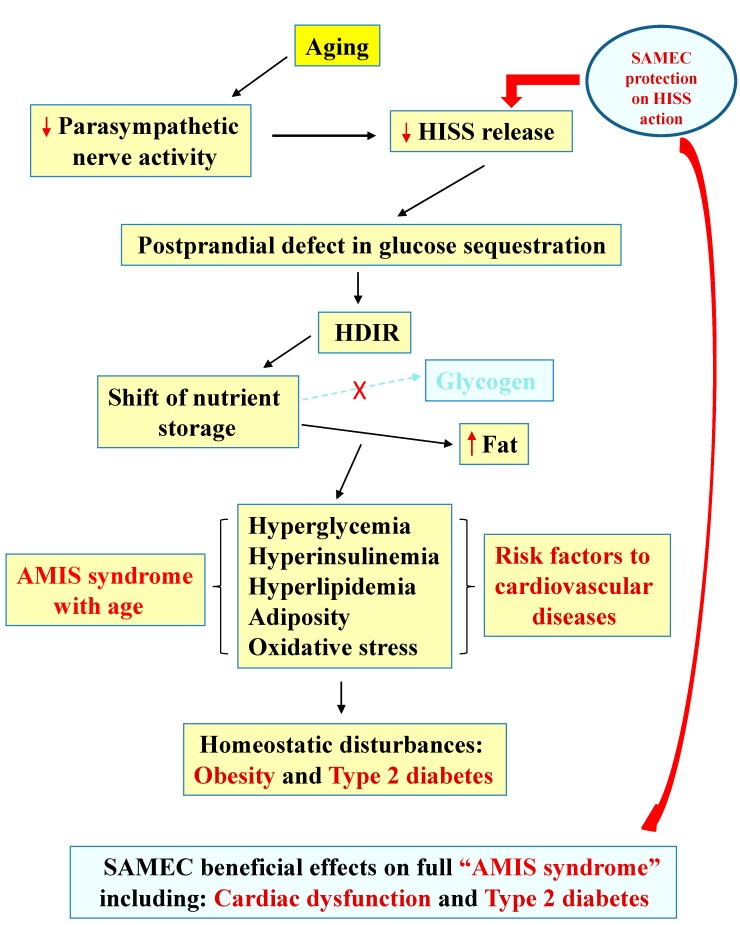
The mechanistic pathway of age-related development of HDIR, AMIS syndrome and antioxidant protection.

Aging results in progressive reduction of insulin-induced HISS release leading to the AMIS syndrome (Figure 2). HISS and insulin action can be separately quantified [45] and show distinctly different patterns of dysfunction, with the development of HDIR occurring as the initiating event and resistance to insulin appearing later in the AMIS syndrome, after the appearance of adiposity and many metabolic and cardiovascular dysfunctions.

The role of free radicals and antioxidants in metabolic regulation is not clear. The etiology of reduced parasympathetic nerve action with age and with the diet-induced models of AMIS is unknown. Much of the inconsistency in demonstration of beneficial effects of antioxidant supplements is possibly related to the observation that powerful synergies exist between antioxidants that act by different mechanisms. The combination of *S*-adenosylmethionine, vitamin E and vitamin C (but not the individual components) is able to suppress the development of HDIR. This cocktail prevented or greatly inhibited the reduced HISS action, preserving MIS and preventing the AMIS syndrome, with beneficial effects shown for both metabolic and cardiovascular function. If adequate antioxidants cannot be provided with the diet, use of SAMEC should be considered as a means of avoiding many of the devastating effects of aging.
